# Origins of mammalian vertebral function revealed through digital bending experiments

**DOI:** 10.1098/rspb.2024.0820

**Published:** 2024-07-10

**Authors:** Katrina E. Jones, Kenneth D. Angielczyk, Stephanie E. Pierce

**Affiliations:** ^1^ Museum of Comparative Zoology and Department of Organismic and Evolutionary Biology, Harvard University, 26 Oxford Street, Cambridge, MA 02138, USA; ^2^ Department of Earth and Environmental Sciences, University of Manchester, Williamson Building, Oxford Road, Manchester M13 9PL, UK; ^3^ Negaunee Integrative Research Center, Field Museum of Natural History, 1400 South Lake Shore Drive, Chicago, IL 60605-2496, USA

**Keywords:** axial skeleton, synapsida, functional morphology, mammal evolution, locomotion

## Abstract

Unravelling the functional steps that underlie major transitions in the fossil record is a significant challenge for biologists owing to the difficulties of interpreting functional capabilities of extinct organisms. New computational modelling approaches provide exciting avenues for testing function in the fossil record. Here, we conduct digital bending experiments to reconstruct vertebral function in non-mammalian synapsids, the extinct forerunners of mammals, to provide insights into the functional underpinnings of the synapsid–mammal transition. We estimate range of motion and stiffness of intervertebral joints in eight non-mammalian synapsid species alongside a comparative sample of extant tetrapods, including salamanders, reptiles and mammals. We show that several key aspects of mammalian vertebral function evolved outside crown Mammalia. Compared to early diverging non-mammalian synapsids, cynodonts stabilized the posterior trunk against lateroflexion, while evolving axial rotation in the anterior trunk. This was later accompanied by posterior sagittal bending in crown mammals, and perhaps even therians specifically. Our data also support the prior hypothesis that functional diversification of the mammalian trunk occurred via co-option of existing morphological regions in response to changing selective demands. Thus, multiple functional and evolutionary steps underlie the origin of remarkable complexity in the mammalian backbone.

## 1. Introduction

Among tetrapods, mammals are distinguished by a suite of evolutionary innovations linked with a highly active lifestyle, such as endothermy and asymmetric gaits [1]. The sequence and timing of the evolution of mammalian traits are recorded in the rich fossil record of non-mammalian synapsids (NMSs) [2–4], the extinct forerunners of mammals, but inferring complex behaviours such as locomotion from fossilized skeletons is very challenging [5,6]. Although fossil trackways can provide a glimpse of once dynamic movements (e.g. [7]), the trackmaker is typically unknown and footfall patterns often fit multiple plausible gaits scenarios [8,9]. Increasingly, palaeontologists use digital modelling as a tool for understanding function in extinct species [8,10–14], providing clues to the evolutionary steps underlying major functional transitions [15–17]. Here, we apply digital modelling to the vertebral column of NMSs for the first time to our knowledge, to gain insights into the origins of the mammalian axial skeleton and its morphofunctional diversity.

The synapsid–mammal locomotor transition has been characterized as a shift from abducted (sprawling) to adducted (upright) limb postures and gaits [17]. As extant lizards with abducted limbs use extensive lateral (side-to-side) bending of the backbone, and extant mammals use sagittal (up-down) bending during ‘asymmetric’ running gaits, it has long been assumed that the synapsid–mammal locomotor transition is also typified as a shift from lateral–sagittal vertebral motions. Of note, the monotreme echidna employs an ‘upright sprawling’ gait that involves rocking of the trunk from side-to-side [18], a behaviour also proposed for pareiasaurs and other Palaeozoic tetrapods [19]. However, recent quantitative analysis of vertebral shape across amniotes revealed distinct vertebral anatomies in early synapsids compared to modern-day reptiles and mammals, and that the synapsid spine was ancestrally stiff rather than laterally compliant [20]. Further, extant mammals display both sagittal bending posteriorly and axial rotation anteriorly, with important morphological features associated with these functions first arising in probainognathian cynodonts, close to the origin of mammals and before the appearance of monotremes [21]. Thus, our understanding of synapsid locomotor evolution is more multi-faceted than previously appreciated, and a detailed investigation of vertebral function is required to tease apart the steps that underlie its origin.

The evolution of well-defined dorsal or ‘trunk’ regions (between the neck and sacrum) in mammals has also been widely linked to physiological and locomotor shifts, such as diaphragmatic breathing and asymmetric gaits [2,3,22,23]. However, it is now clear that some degree of trunk regionalization is ubiquitous across amniotes (if not vertebrates) [24,25], and that regionalization patterns in mammals are more elaborate than the traditional thoracic–lumbar division allows, with statistical evidence for at least four regions between the neck and the sacrum [22,26]. Furthermore, biomechanical analysis of vertebral columns in extant mammals and lizards suggests that novel functions are probably the result of co-option of existing morphological regions, not the addition of new ones [21]. Thus, the relationship between vertebral function, regionalization and synapsid locomotor evolution remains unresolved.

The recent flurry of work on synapsid vertebral morphology has demonstrated that axial evolution over the synapsid–mammal transition involves multiple steps, but the implications for functional evolution are unclear. Functional inferences in previous studies were based on simple morphofunctional metrics that could be applied to a broad range of taxa and summarized across many vertebrae [20]. By contrast, digital modelling provides a unique opportunity to dissect the craniocaudal patterns in joint function across the entire vertebral column within individual taxa [11,14,15,27,28]. Although this approach is more time consuming than traditional metrics-based inferences and requires a much higher degree of preservation of the vertebrae, it provides unparalleled detail and the ability to test hypotheses about the functional impact of certain morphological features [17]. Joint mobility estimation from digital models has been applied before (see the introduction of [11] for more discussion), but recent application of automated approaches also provides the ability to account for model uncertainty by exploring the parameter space, such as the influence of soft tissues [11,13]. Advances in digital modelling thus creates an opportunity to address the question of synapsid vertebral functional evolution more precisely.

In this study, we apply AutoBend, a semi-automated digital modelling approach that estimates range of motion (ROM) of intervertebral joints based on bony morphology and the inferred impact of soft tissues. Using three-dimensional models based on osteology alone, AutoBend has been shown to replicate *ex vivo* experimentally derived ROM patterns and magnitude at intervertebral joints in both extant mammals and reptiles [11]. Here, we estimate intervertebral joint ROM and stiffness in the trunk vertebrae of seven extant tetrapods and eight exceptionally preserved extinct NMSs, with an emphasis on crownward therapsids and cynodonts. We show that these advanced synapsids share some of the key functional traits that characterize crown mammals, allowing us to reconstruct the evolutionary sequence of synapsid vertebral functional evolution.

## 2. Methods

### (a) Specimens

Our sample includes the three major grades of NMS, including two ‘pelycosaurs’, three non-cynodont therapsids and three non-mammalian cynodonts (electronic supplementary material, table S1). Only fossils with minimal taphonomic distortion and greater than 50% complete vertebral columns were used in this study (electronic supplementary material, table S2). For comparison, we also selected seven representative extant tetrapods (electronic supplementary material, table S1). These include a salamander (extant outgroup to living amniotes), three reptiles and three mammals. Within reptiles, an archosaur (crocodile) and two lepidosaurs were selected (tegu, tuatara); the tuatara shares morphological similarities with NMSs, including amphicoelous vertebrae and intercentra [20,29]. Within mammals, we sampled to maximize phylogenetic and functional diversity. A monotreme (echidna) was selected, representing the most basally branching group of living mammals, as well as two therians, including one ‘stiff backed’ species (sheep, artiodactyl, transverse gallop) and one with extensive intervertebral compliance (cat, carnivoran, rotary gallop). All extant specimens were fully mature adults that were free of pathology. Three-dimensional models of the vertebral columns of each species were generated through micro-computed tomography (CT) or surface scanning as part of an earlier study, and are described elsewhere [22]. Our study focuses on the trunk vertebrae (first thoracic/dorsal joint to first sacral joint inclusive) because this is the region most involved in terrestrial quadrupedal locomotion [30,31].

### (b) Model preparation

For both extant and extinct taxa, individual trunk vertebrae were digitally rearticulated following the steps outlined by Jones *et al.* [11]. In 3Matic (Materialise) software, vertebrae were aligned so that vertebral centra were parallel to one another and zygapophyseal joints were maximally overlapping. The centre of rotation (COR) of each joint was defined as the midpoint of the intervertebral space between adjacent amphicoelous/acoelous centra (mammals, salamander, tuatara, NMS) or the geometric centre of a sphere fit to procoelous centra (crocodile, tegu). Once aligned, vertebrae and CORs were imported into Autodesk Maya 2023, and models built using the following steps: (i) set rotation axes, (ii) place locators for soft-tissue constraints, and (iii) hierarchically link joints ready for rotation about COR [11]. The Python code used to set up the vertebral model is freely available on github (https://github.com/katrinajones/AutoBend).

Axes of rotation for each joint were set as follows: X, axial rotation; Y, lateral bending; Z, sagittal bending. Boolean objects were created for each joint that measure the degree of overlap between adjacent vertebrae to estimate bony intersection. Locators were placed at the following positions: dorsal, ventral and lateral extremes of the centrum endplate; cranial and caudal extremes of the pre- and post-zygapophysis [11]. These locators reflect the major ligamentous connections of the joint (intervertebral disc, zygapophyseal capsule) and the soft-tissue constraints on motion were implemented based on their relative position. Finally, all the objects in the model (vertebrae, Boolean objects, rotation axes and soft-tissue locators) were parented such that rotation at each joint moves all objects that are caudal to that joint axis.

### (c) Range of motion and stiffness

To calculate intervertebral ROM, digital bending experiments were run by batch applying AutoBend to the Maya models using MayaPy, an external python interpreter for Autodesk Maya. AutoBend code is freely available on github (https://github.com/katrinajones/AutoBend), and the MayaPy code is provided as the electronic supplementary material (*Wrapper_vert_bending-synapsids.py*). For each joint, AutoBend rotated the posterior vertebra about the COR in six bending directions (left rotation, right rotation, left lateroflexion, right lateroflexion, dorsoflexion, ventroflexion) in half-degree increments until a constraint on motion was reached. Joint translations were not included as they had minimal impact on motion in a validation study [11]. Joint spacing was varied to account for uncertainty in model reconstruction (optimal spacing ± 10%) [11,32].

Constraints on motion included bony intersection, displacement at the centrum (modelling strain on ligaments of the intervertebral disc) and displacement at the zygapophyses (modelling strain in the capsular ligaments) [11]. Bony intersections were permitted a small ‘intersection threshold’, or overlap, to enable joints to slide past one another more smoothly. Intersection threshold was varied by 0.25%/0.5%/0.75% of average vertebral area to account for inflation of the model while wrapping or smoothing. Strain (length change along a given axis) was limited at both the centra and zygapophyses based on the position of the soft-tissue locators. The degree of strain permitted was 50% of the original distance between the locators and was varied by ±10% to account for uncertainties in soft tissue properties. It is worth noting that the AutoBend models specifically consider bone–bone interactions and the impact of the discs and zygapophyseal ligaments. There are other soft-tissues that may also impact mobility of the spine, for example, epaxial and extrinsic shoulder musculature and interspinous ligaments. These tissues are much more difficult to account for in the fossil record owing to uncertainty in myology and frequent damage to spinous processes. Total ROM in each bending direction, as well as the constraints limiting motion, were exported as a text file for each joint for all 27 parameter variants.

Relative intervertebral stiffness was estimated using both ROM (above) and morphological measurements reflecting soft-tissue dimensions impacting joint stiffness (e.g. disc dimensions, see [11] for full discussion). While ROM and stiffness are not independent of one another, including stiffness allows us to explicitly account for important morphofunctional variation not captured by ROM alone. Relative stiffness at each intervertebral joint was determined using calcStiffness from the AutoBend plotting functions, estimated as the ratio of bending moment to angular displacement (ROM) and size-corrected based on average centrum length following [8]. Bending moment was approximated as the lever arms in sagittal (linear distance from the COR to the top of the vertebral arch) and lateral bending (linear distance from the COR to the lateral edge of the centrum), scaled by centrum area [11].

### (d) Structure-removal analysis

‘Pelycosaurs’ exhibit some unusual vertebral features whose function is unknown, including a dorsal ‘sail’ composed of hyperelongate neural spines and small intercentra located between large pleurocentra (the functional vertebrae). To examine the impact of these structures on vertebral function, models were created with and without these features and intervertebral ROM calculated. The impact of hyperelongate neural spines was assessed using the exceptionally preserved sail in *Edaphosaurus* (DMNH 20110401). Duplicate models were created with the complete sail intact, or with the neural spines removed just above the vertebral arch. To assess the impact of intercentra in a reptile and a ‘pelycosaur’, four models were created: tuatara with intercentra, tuatara without intercentra, *Edaphosaurus* with intercentra, and *Edaphosaurus* without intercentra. The *Edaphosaurus* specimen was preserved with most of the dorsal intercentra *in situ*. For some joints, the intercentra were displaced from the joint, and so were realigned with the ventral margin of the centrum. The tuatara specimen was scanned with the vertebral column in articulation and intercentra in place, providing the opportunity to model these elements precisely in their anatomical position. For the models with intercentra, the three-dimensional mesh of the intercentrum for each joint was combined with the three-dimensional mesh of the anterior vertebra to produce a single object, about which the posterior vertebra was rotated during bending. For the models without intercentra, the intercentra were deleted from the model.

### (e) Data analysis

ROM data were processed and visualized in R using custom code available in Autobend (*Autobend_Plotting_Functions.R*). AutoBend data exported from Maya were loaded into R using *load.insilico* from the AutoBend plotting functions. Axial rotation and lateroflexion were averaged across right and left bending directions to account for differences in symmetry and then multiplied by two to ensure values remained on the same scale. However, if joints were asymmetrically damaged, results from the intact side only (left or right) were used to calculate ROM in axial rotation and lateroflexion, and those values doubled. The constraints that limited motion during each iteration of the AutoBend analysis were summarized for each species and bending direction using bar plots in *ConstonMotion* (AutoBend plotting functions). The 0.75% bony intersection threshold repeats in all directions were removed *a posteriori* as many of the intervertebral joints had disarticulated in axial rotation prior to reaching this threshold. The percentage contribution of each bending direction ROM to total ROM for each intervertebral joint was summarized in a ternary plot, using the function *ggtern* in *ggplot2*. Patterns of ROM and stiffness along the vertebral column were visualized using scatterplots of values against vertebral position, grouped by bending direction, using *craniocaudPlot* (AutoBend plotting functions).

### (f) Statistics

A multivariate analysis of variance (MANOVA) was used to test for significant differences in vertebral function between reptiles and mammals, as well as among the sampled species, using the model: y ~ Group/Species * Region. Tests were conducted on joint-species mean values (averaged across repeats of the experiment, *n* = 219) of ROM (lateral, sagittal and axial rotation) and stiffness (lateral and sagittal bending). Vertebral joints were grouped into region (anterior/posterior) based on either the position of the diaphragmatic vertebra (mammals) or the mid-dorsal position (reptiles). We modelled species as a nested factor within clade/grade groups (electronic supplementary material, table S1), instead of using a phylogenetically corrected analysis (e.g. phylogenetic generalized least squares (PGLS)), because the species sample was small and this approach enabled inclusion of multiple observations for each species, e.g. from anterior and posterior joints. MANOVA’s were conducted using lm.rrpp in the R package rrpp using randomization of residuals in a permutation procedure with 10 000 iterations [33]. This non-parametric method uses empirical probability distributions to reconstruct model effects when using multiple dependent variables more reliably. Similarly, RRPP-MANOVA was used to assess the impact of hyperelongate neural spines and intercentra on ROM, with crossed factors of species and model (with/without structure).

## 3. Results

### (a) Patterns of intervertebral joint range of motion

**Figure 1.**
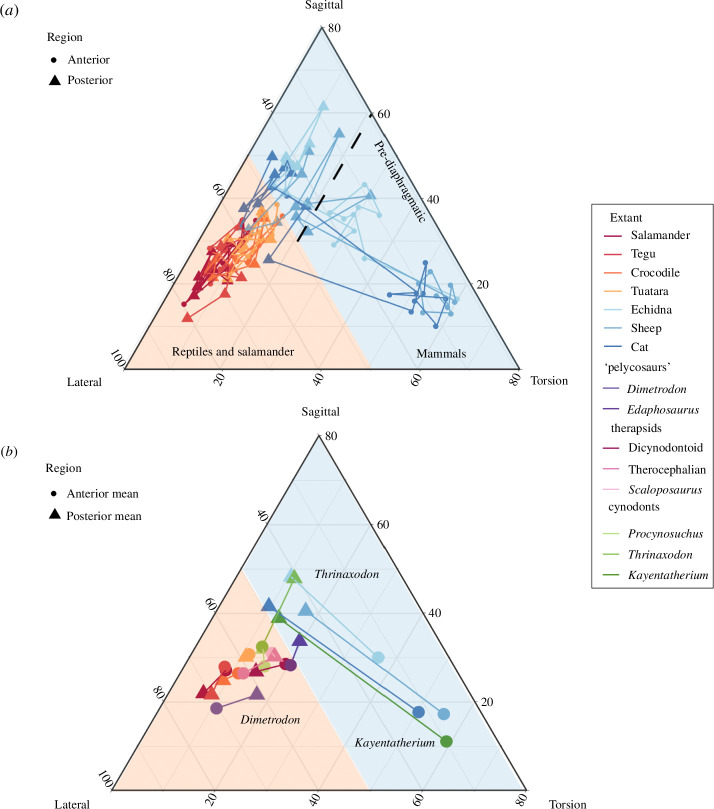
Ternary plot of intervertebral joint ROM for (*a*) extant (all joints) and (*b*) extant and extinct taxa (anterior and posterior mean). Joints that plot close to the vertices of the ternary are specialized for one type of motion, whereas those nearer the middle have mobility in multiple directions. Axes represent mobility as a proportion of overall mobility and are truncated in sagittal bending and axial torsion to eliminate empty plot space. Blue shaded; sagittal–lateral bending proportion greater than 50%; orange shaded, sagittal–lateral bending proportion less than 50%. For individual species plots, see the electronic supplementary material S1 and S2.

The ternary plot depicts the proportion of intervertebral joint ROM in each bending direction (lateral, sagittal and axial) compared to total ROM for each joint (figure 1). In extant taxa, there are distinct mobility patterns for mammals relative to reptiles/salamanders (figure 1*a*; electronic supplementary material, figure S1), which are reflected in a significant difference between mammals and reptiles in the MANOVA analysis (electronic supplementary material, table S3; *p* = 0.016, *R* ^2^ = 24%). The reptile and salamander joints tend to cluster closer to the lateral vertex of the ternary, indicating an emphasis on lateroflexion. By contrast, the mammal taxa have divergent vertebral functions in anterior and posterior intervertebral joints, as indicated by a significant effect of region in MANOVA (electronic supplementary material, table S3; *p* = 0.001, *R* ^2^ = 19%). Anterior joints are dominated by high mobility in axial rotation, whereas posterior joints are either equally weighted in sagittal and lateral bending or favour sagittal bending. NMSs exhibit a range of functions that spans both mammal and reptile/salamander space (figure 1*b*; electronic supplementary material, figure S1). The ‘pelycosaur’ *Dimetrodon* emphasizes strong lateral bending, in a pattern that closely resembles extant reptiles. *Edaphosaurus,* the three therapsid taxa, and *Procynosuchus* all occupy a space that is intermediate between the reptile/salamander and mammal cluster, indicating that lateroflexion makes up around 50% of their functional repertoire. *Thrinaxodon* and *Kayentatherium* exhibit more mammal-like patterns (figure 1*b*; electronic supplementary material, figure S2). The anterior joints of *Thrinaxodon* cluster with the other NMSs, whereas the posterior joints display a lateral–sagittal ratio within the mammal space, clustering with the echidna. *Kayentatherium* displays a very therian-like mobility profile. Anterior joints display high axial rotation, whereas the posterior joints have a lateral–sagittal ratio most comparable to the cat and sheep. Examining the structures which most commonly restrict motion in each taxa can reveal the impact of specific morphologies on ROM (electronic supplementary material, figure S3). Results reveal that bony interactions and disc strain impact mobility in lateroflexion, while sagittal bending is often limited by compression or tension in the zygapophyseal joints.

**Figure 2.**
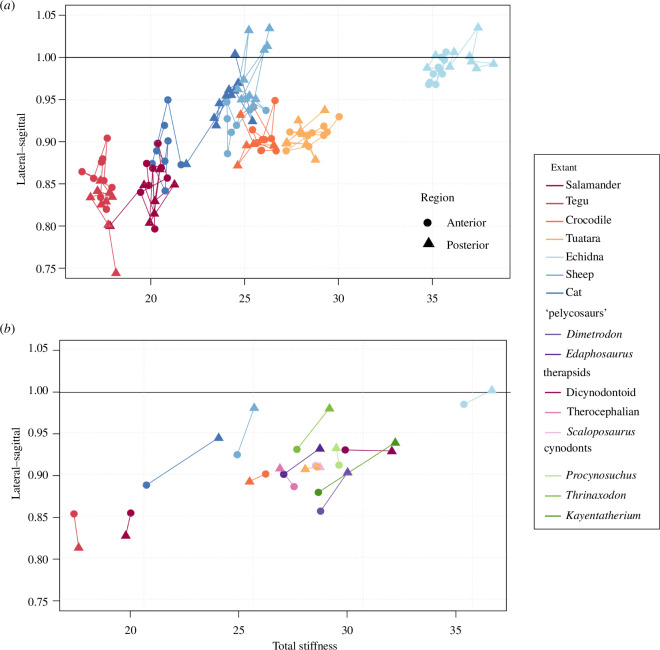
Lateral–sagittal stiffness ratio against total relative stiffness (summed lateral and sagittal stiffness). (*a*) extant taxa (all joints) and (*b*) extant and extinct taxa (anterior and posterior mean). Black line, stiffness equal in lateral and sagittal plane.

### (b) Influence of neural spines and intercentra

There was no significant effect of neural spine removal on mobility (electronic supplementary material, table S4; *p* = 0.328), suggesting that the hyperelongate neural spines of the dorsal ‘sail’ in *Edaphosaurus* did not limit ROM. Removal of the intercentra resulted in a marked increase in sagittal bending in the dorsal intervertebral joints of the tuatara (electronic supplementary material, figure S4*a,b*). This result was confirmed by the MANOVA (electronic supplementary material, table S4), where there was a significant effect of intercentra, though the effect size was quite small (*R* ^2^ = 9%). Examination of craniocaudal patterns in mobility between models with and without intercentra suggest that they significantly limit ventroflexion in the tuatara (electronic supplementary material, figure S4). The impact of intercentra was more subtle in *Edaphosaurus,* with only a small increase in sagittal bending following their removal (electronic supplementary material, figure S4). These differences are confirmed by the significant interaction of species and intercentra in the MANOVA, indicating that the impact of removing intercentra varies between the two species (electronic supplementary material, table S4).

### (c) Patterns of intervertebral joint stiffness

We estimated intervertebral joint stiffness using mobility and vertebral morphology [11]. This variation was summarized by plotting the ratio of lateral to sagittal stiffness against the total relative stiffness (sum of lateral and sagittal) for all the joints (figure 2*a*). Most joints were more compliant in lateral than sagittal bending (stiffness ratio <1). However, as the stiffness ratio approached one (equal in both directions), the total stiffness increased, suggesting more stiffness in the lateral plane. In general, mammals tended to have a stiffness ratio above 0.9, except for the anterior joints in the cat, which were highly compliant (electronic supplementary material, table S3). The crocodile and tuatara hover around a ratio of 0.9, and the tegu and salamander fall below that level, indicating compliance in lateroflexion. This divergence in properties among reptiles/amphibians is reflected in the highly significant effect of species in the MANOVA (electronic supplementary material, table S3; *R* ^2^ = 84%, *p* = 0.001), but insignificant effect of group (electronic supplementary material, table S3; *R* ^2^ = 0.6%, *p* = 0.5). All the NMSs examined showed high overall stiffness and stiffness ratios of 0.9 or above (figure 2*b*), suggesting they are relatively stiff in both lateroflexion and sagittal bending. Mammals also tend to have higher stiffness in posterior than anterior intervertebral joints, indicating a caudal increase in stiffness along the column (figure 2*b*, positively directed vector between anterior and posterior means), a finding which is supported by *ex vivo* experiments [21,34]. Most of the NMSs, notably *Thrinaxodon* and *Kayentatherium,* share this pattern.

### (d) Functional evolution and regionalization

**Figure 3.**
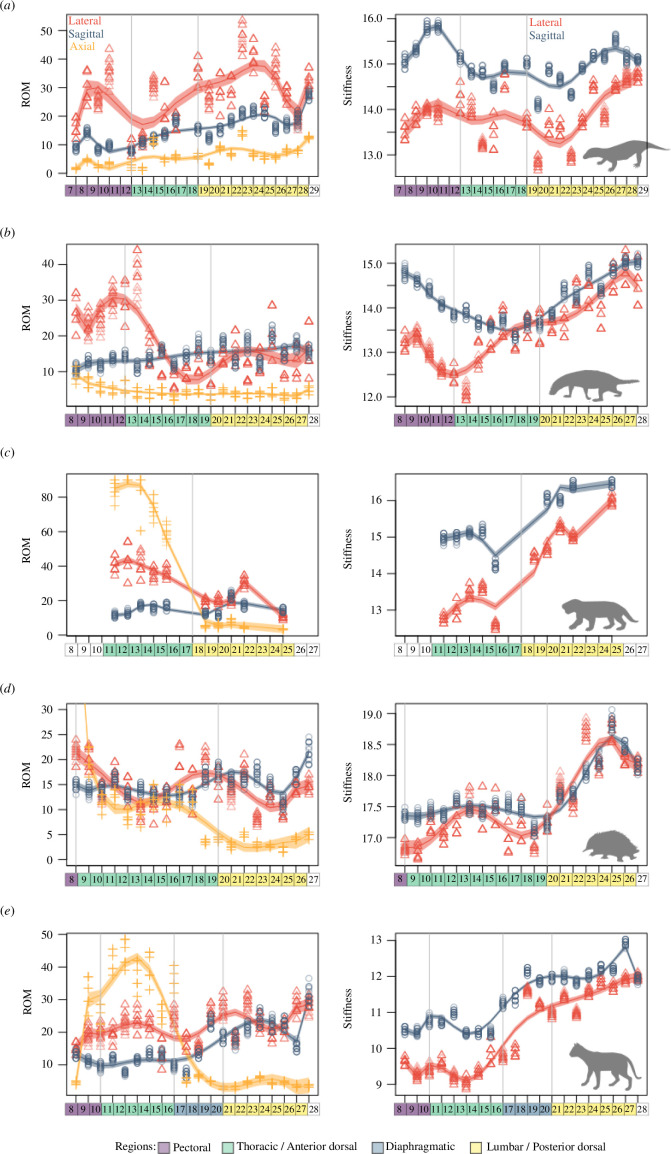
Comparison of mobility and stiffness patterns with vertebral regionalization in the trunk region (first thoracic/dorsal to first sacral joint inclusive) across the cynodont–mammal boundary. (*a*) *Procynosuchus delharpeae*, (*b*) *Thrinaxodon liorhinus*, (*c*) *Kayentatherium wellesi*, (*d*) *Tachyglossus aculeatus,* and (*e*) *Felis catus*. Joint numbers are counted post-cranially and are named for the anterior vertebra in the joint (e.g. joint 8 is the joint between post-cervical-7 and post-cervical-8). ROM for lateral bending and axial rotation were averaged across left and right sides then multiplied by two, representing an estimate of full amplitudes of motion. Craniocaudal patterns represented by polynomial splines. Echidna ROM data (*d*) is truncated at 30° to allow better visualization of lateral and sagittal bending, however, axial rotation at the first joint reaches the maximum allowed 90°. ROM and stiffness from AutoBend models, and regionalization patterns based on vertebral morphology from Jones *et al*. [16]. ROM and stiffness are intended to be viewed comparatively, as opposed to absolute values. Grey vertical lines indicate boundaries between vertebral regions. For full comparison of craniocaudal patterns in all sampled taxa, see the electronic supplementary material, figures S5–S9.

To delve more deeply into the evolutionary patterns underlying the origin of mammalian vertebral function, we compared craniocaudal patterns of ROM and stiffness across the cynodont–mammal boundary (figure 3). Region boundaries are based on morphological regions from Jones *et al*. [22]. Representing a crown therian, the cat displays a peak in axial rotation in the thoracic region (figure 3*e*: purple), increasing sagittal bending and to a lesser extent lateroflexion in the lumbar region (figure 3*e*: yellow), and higher overall stiffness in the diaphragmatic and lumbar regions (figure 3*e*: blue, yellow). Similar patterns are also seen in the sheep, the other therian in the sample (electronic supplementary material, figure S5). By contrast, axial rotation in the echidna is limited to the first thoracic joint owing to the development of enlarged metapophyses on the other thoracic vertebrae (figure 3*d*: purple). Lateral and sagittal bending are generally limited in the echidna, with ROM below 20° and the peak in sagittal bending restricted to the lumbosacral joint. However, there is a large increase in stiffness in the posterior trunk as seen in the cat (figure 3*d*: yellow). Examining the non-mammalian cynodonts sampled here reveals the acquisition of mammalian vertebral function to varying degrees. The basal cynodont *Procynosuchus* displays the ancestral condition for synapsids, with an emphasis on lateral over sagittal bending across the entire dorsal column (figure 1), but with some variation in mobility and stiffness among the trunk regions (figure 3*a*). However, there is greater functional divergence between anterior and posterior regions in the more advanced cynodonts *Thrinaxodon* and *Kayentatherium*. In *Thrinaxodon,* lateroflexion remains high in the anterior column but there is a distinctive reduction in lateral bending in the posterior column (figure 3*b*). Furthermore, *Kayentatherium* exhibits high levels of axial rotation in the anterior dorsal region that are characteristic of crown mammals (figure 3*c*).

## 4. Discussion

The vertebral column is a key component of the mammalian locomotor apparatus, but its evolution remains largely understudied relative to the limbs because of its anatomical complexity. Recent advances in digital modelling have made detailed assessment of vertebral function from osteological or fossil specimens feasible [11–13,15,16,35]. Here, we applied AutoBend, an experimentally validated digital bending algorithm [8], to quantify intervertebral joint function in extinct NMSs. We show that key elements of the mammalian axial functional repertoire evolved in the NMS lineage prior to the crown group; and find support for the hypothesis, based on extant taxa, that functional innovations in the synapsid vertebral column were achieved by co-option of existing vertebral regions for new functions [21]. Further, we demonstrate the role of intercentra, plesiomorphic vertebral structures found in basal synapsids, in vertebral stabilization and test the impact of the dorsal ‘sail’ on mobility.

### (a) Key mammalian vertebral functions evolved in cynodonts

The mammalian trunk is capable of a remarkable diversity of motions relative to those of other amniotes [21]. *Ex vivo* studies show that although other quadrupedal tetrapods (e.g. salamanders, lizards, crocodilians) primarily engage in lateral bending of trunk vertebral joints, some mammals use mobility in lateroflexion, sagittal bending and axial rotation [21,34,36,37], reflecting their varied behaviour and ecology. However, owing to the paucity of vertebral biomechanical data, little is known about how these mechanical functions vary among living mammals, or when they first appeared in the synapsid lineage. Using AutoBend [11], we estimated intervertebral joint mobility and stiffness in a range of modern tetrapods and fossil NMSs to dissect evolutionary variation in vertebral function and the morphological traits that underlie it.

Twisting of the spine is generally limited in non-mammal tetrapods with sprawling gaits to stabilize against torsional loads during locomotion [38]. However, high levels of axial rotation have been described *ex vivo* in the thoracic intervertebral joints of cats, suggesting that this is a distinctive mammalian function [21,34]. How this type of mobility may manifest *in vivo* is unknown in most mammals, though well-described in humans [39]. There are hypothesized links to behaviours such as grooming, curling up or manoeuvring during locomotion, and the epaxial muscles act to resist this motion during straight-line walking and trotting [20,21,30,38,40]. Furthermore, axial rotation in echidnas has been linked to their unique ‘upright sprawling’ walking gait in which the body rocks from side to side during locomotion [18].

The capability for axial rotation in vertebral joints relates to the unique morphology of the thoracic zygapophyses. Specifically, horizontally oriented ‘pre-diaphragmatic’ joints are hypothesized to permit axial rotation through their more ‘open’ articulation that limits bony intersection. Among the extant mammals sampled here, all share axial rotation at some or all of the pre-diaphragmatic joints (figure 3; electronic supplementary material, figure S5−S9), supporting the hypothesized link between form and function. Furthermore, we confirm axial rotation in the probainognathian cynodont *Kayentatherium* [21], corresponding with the earliest appearance of mammal-like pre-diaphragmatic joints in the synapsid fossil record (figure 1; electronic supplementary material, figure S2). However, we also find evidence for secondary stabilization against axial rotation in some mammals. In the echidna, T1–T2 exhibited significant axial rotation as in the other mammal species examined, but twisting was restricted in the more caudal pre-diaphragmatic joints by the presence of large metapophyses (figure 3*d*). Enlarged metapophyses evolved repeatedly in several fossorial mammal groups (e.g. armadillos) and it is hypothesized that this is a secondary loss of mobility in these joints, perhaps relating to ecological specialization [34]. This also suggests that the ‘upright sprawling’ gait of echidnas, along with other aspects of their unusual morphology (e.g. pes), may be a derived trait and not the ancestral condition as indicated by some mechanical models [19].

**Figure 4.**
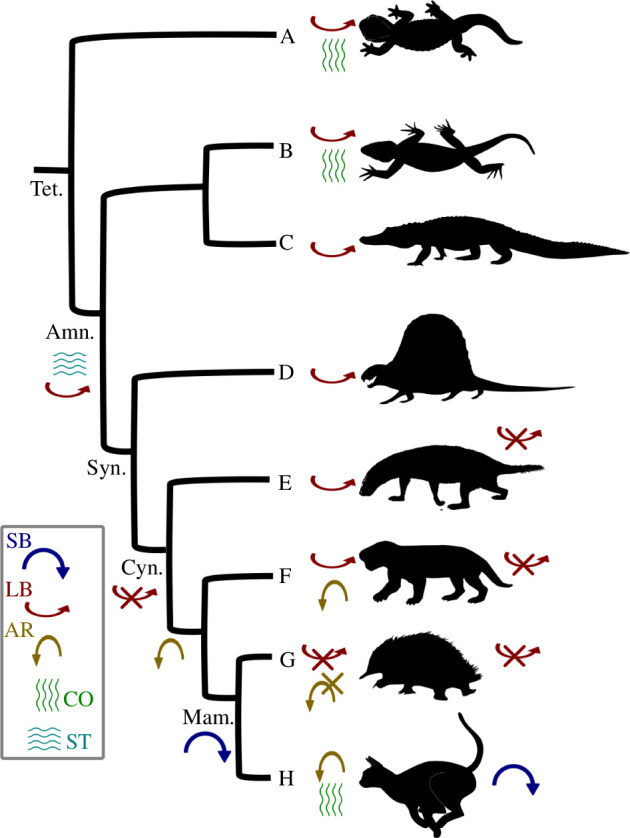
Summary of functional evolution of the synapsid vertebral column. SB, sagittal bending; LB, lateral bending; AR, axial rotation; CO, compliance; ST, stiffness. A, salamander; B, tegu; C, crocodile; D, *Dimetrodon*; E, *Thrinaxodon*; F, *Kayentatherium*; G, echidna; H, cat.

Sagittal movements in the posterior thoracic and lumbar joints of mammals are linked with the evolution of fast locomotion because sagittal bending occurs at these joints during asymmetric gaits [38,41]. Our data recover a peak in sagittal bending in the lumbar region and a second larger peak at the lumbosacral joint in both the cat and sheep (figure 3*e*; electronic supplementary material, figure S5), supporting *in vivo* and *ex vivo* biomechanical experiments [21,34,41]. However, our data also provide new insights into how this important mechanical function may have evolved. Figure 1 demonstrates that the cynodonts *Thrinaxodon* and *Kayentatherium* exhibit higher ratios of sagittal–lateral mobility in the posterior joints compared to other NMSs, like those of extant mammals. However, closer inspection of figure 3 reveals that this consists of two separate traits. While these cynodonts exhibit high levels of lateroflexion anteriorly, their posterior joints show lateral stabilization without the mammalian peak in sagittal bending (figure 3*b,c*; electronic supplementary material, figure S9). The echidna has relatively limited lateral bending and sagittal bending throughout the trunk, except at the lumbosacral joint, without the distinctive mobility patterns or morphology observed in most mammalian lumbar regions [22]. Thus, posterior sagittal bending appears to be a therian attribute, with both the cat and sheep characterized by high levels of sagittal bending in the lumbar vertebrae and especially at the lumbosacral joint (figure 3*e*; electronic supplementary material, figure S5). Therefore, we hypothesize that stabilization against lateroflexion evolved first in the trunks of non-mammalian cynodonts, followed later by enhanced sagittal bending in therian mammals (figure 4).

Carrier [42] suggested that stabilization of the vertebral column against lateroflexion in cynodonts was a key step in the evolution of ‘upright’ postures and the decoupling of breathing and locomotion in synapsids. ‘Carrier’s constraint’ posits that sprawling tetrapods are restricted from running and breathing at the same time owing to the dual function of the trunk and abdominal musculature in both behaviours. Thus, greater capacity for locomotor stamina was unleashed in the mammalian lineage by decoupling the muscles used for breathing from those used for locomotion, and this is correlated with the evolution of diaphragmatic ventilation [3,42]. Carrier [43] posited that passive stabilization of the trunk against lateroflexion via fused/imbricating ribs in cynodonts would free up the lateral trunk muscles to assist in breathing. Our result supports this contention by demonstrating that the construction of the intervertebral joints in cynodonts also contributes to passive lateral stabilization (figure 3). Therefore, we hypothesize that the lateral stabilization observed here relates to shifting ventilatory mechanisms, increased metabolic capacity and higher activity in cynodonts, as opposed to the origin of sagittal mobility or asymmetric gaits [42]. This agrees with a breadth of other evidence suggesting increased activity and metabolism in cynodonts [2,23,44], as well as increased overall complexity of the vertebral column [45].

Stiffness of the backbone is linked with stabilization against gravity and elastic energy storage during locomotion [21,34]. We find high overall stiffness in NMSs relative to therian mammals and some reptiles and amphibians (e.g. salamander and tegu) [20], but similar to the tuatara and crocodile (figure 2). This observation supports morphometric data suggesting a relatively stiff vertebral column may be ancestral for amniotes, and should be further explored across ‘pelycosaurs’ and other Palaeozoic tetrapods of varying sizes (although it is unlikely that even the smallest species would be exact locomotor analogues of modern taxa given their divergent morphology) [20]. Notably, the anterior joints of therian mammals, especially the cat, are highly compliant compared to the other synapsid taxa examined (figure 2). This may be a secondary adaptation to facilitate forelimb mobility during climbing or handling prey but requires further investigation. However, the general pattern of stiffness increasing caudally along the column holds across the sampled mammals and most of the NMSs (figure 2; electronic supplementary material, figures S5–S9). It has been hypothesized that the posterior trunk in mammals may function as a ‘stiff but mobile’ zone which results in passively stable joints that can still be activated under high loads (e.g. during locomotion) [21,34].

Figure 4 summarizes the overall evolutionary patterns in vertebral function observed here alongside the literature. We suggest that the trunk of amniotes was ancestrally stiff, with lateral mobility intermediate between that of extant mammals and some reptiles. The evolution of mammalian trunk function occurred in several stages: first, the posterior column in cynodonts was stabilized against lateral bending to facilitate locomotor–respiratory decoupling (figure 4, red arrow with cross). Second, axial rotation in the anterior trunk appears in advanced cynodonts, associated with reorientation of the pre-diaphragmatic zygapophyses and perhaps the origin of mammalian behaviours such as grooming (figure 4, gold arrow). Finally, the origin of enhanced lumbar sagittal mobility probably did not occur until within the mammalian crown, and potentially even therians specifically, in association with specialization for running gaits (figure 4, blue arrow). Examination of mammaliaforms and stem therians could help to disentangle how and when this final step occurred, or if it is a specific adaptation of cursorial therians.

### (b) Co-option of vertebral regions underlies functional diversification

Regionalization is the subdivision of the vertebral column into semi-autonomous morphological and/or functional units [22,25,46,47]. In mammals, *ex vivo* experiments have found a close relationship between the boundaries of morphological regions and craniocaudal patterns of intervertebral joint function [21] suggesting a strong link between regionalization and functional differentiation. However, this relationship appears to be decoupled in lizards, where subtle morphological regions in the trunk share similar intervertebral joint function. This result was used to argue that evolutionary diversification in intervertebral joint function in amniotes was achieved by co-option of the modular patterns from pre-existing developmental regions in response to craniocaudal divergence in selective regimes [21,48]. Similarly, the amphibian and the other reptiles examined here exhibited relatively little regional variation in vertebral function across their trunks despite evidence of multiple morphological regions [22]. Tracing these patterns back along the synapsid lineage, basal synapsids also show little functional differentiation of the trunk (figure 3; electronic supplementary material, figures S7−S9), though at least two morphological regions have been recovered for the ‘pelycosaur’ trunk (anterior dorsal and posterior dorsal), supporting ‘cryptic regionalization’ as the ancestral condition for amniotes [25].

This study provides, to our knowledge, the first evidence in support of the co-option hypothesis, that cryptic morphological regions were redeployed for novel and important functions in the synapsid lineage, while maintaining their ancestral modular patterning. In cynodonts, axial rotation evolved in the anterior dorsal region, whereas lateral stabilization appeared in the posterior dorsal region (figures 1*b* and 3*c*), demonstrating the power of underlying modularity in shaping adaptive responses [48]. For example, our data suggest that a pre-existing anterior dorsal region present in all synapsids was co-opted for axial rotation in advanced cynodonts by evolution of a new morphology (horizontal zygapophyses) in those vertebrae specifically. In therian mammals, the posterior dorsal region was further divided into diaphragmatic and lumbar subregions based on the loss of ribs to form a true ‘ribless’ lumbar region [22]. In extant mammals, sagittal bending is often concentrated in the ribless lumbar region [21,31,41], and always limited to the post-diaphragmatic region [41]. Determining if sagittal bending coevolved with either of these traits will require a broader sample of mammaliaforms and stem therian mammals. Therefore, we do not find evidence that regionalization acts as an evolutionary constraint to restrict functional diversification of the vertebral column [49,50]. Instead, the functionally similar regions observed in basal synapsids suggest the vertebral column may be ancestrally ‘over-engineered’, with more morphological regions than required for the functional patterns observed (figure 3). This may relate to the multi-faceted role of vertebral patterning beyond the bony articulations, but also in relation to the broad diversity of other complex structures associated with the vertebral column (ligaments, muscles, nerves etc.) and functions beyond intervertebral mobility and stiffness (e.g. mechanical lever arms for muscles, attenuation). Future work should examine how regionalization patterns map across different structures and elements to understand how coevolution of elements impacts modularity and evolutionary constraint.

### (c) Function of intercentra and hyperelongate neural spines

Within tetrapods, there is large variability in the structure and composition of the elements that make up the vertebral centra [51]. In basal amniotes and synapsids, the pre-sacral vertebrae have a large pleurocentrum and small crescent-shaped ventrally positioned intercentrum. Although the presence of intercentra in pelycosaur-grade synapsids is well documented, the implications for vertebral function are largely unknown. Kemp hypothesized that intercentra were related to the degree of lateral mobility, with their reduction and eventual loss in synapsids indicating the reduction and elimination of lateral bending from synapsid gaits [52]. Our data do not support this hypothesis. Digital bending experiments show that removing intercentra significantly increases mobility in sagittal bending in both *Edaphosaurus* and the tuatara (electronic supplementary material, figure S4) by reducing the degree of bony intersection between vertebrae in ventroflexion but has little impact on lateral bending. This result suggests that intercentra function to stabilize the vertebral column in the sagittal plane. ‘Pelycosaurs’ retained a patent notochord along the vertebral column into adulthood, demonstrated by deeply amphicoelous centra with a notochordal foramen and histological evidence [53]. The biomechanical function of the notochord in early diverging tetrapods is unclear, but intercentra in the vertebral columns of ‘pelycosaurs’ may aid in protecting the patent notochord by stabilizing the dorsal column against shear and ventral bending. Intercentra are lost in therapsids where closure of the notochordal canal indicates a loss of notochordal tissue and an incipient intervertebral disc [54]. The intervertebral disc consists of a proteoglycan nucleus core surrounded by a fibrocartilage annulus ring, which acts to resist tension in the intervertebral joint and thus may supersede the supportive function of the intercentra [55].

Hyperelongate neural spines evolved multiple times in ‘pelycosaurs’, and probably formed an attachment for a sheet of interstitial soft tissue known as a ‘sail’, though its extent and exact anatomy are controversial [4,56,57]. The extreme length of the spines*,* especially in the mid-dorsal region, implies that even a small degree of bending in dorsiflexion could bring the tips of the spines into contact during locomotion and potentially risk damage to these delicate structures. We tested this hypothesis by running our models of *Edaphosaurus* with and without the hyperelongate neural spines. Our results revealed that the spines did not impact on intervertebral mobility, suggesting they do not come into contact during maximum dorsiflexion (electronic supplementary material, figure S4). This suggests that the structure of the intervertebral joint itself restricts motion enough to protect the spines during dorsiflexion. However, this does not imply that sails had no impact on vertebral function. The soft tissues of the sail could not be modelled here, but they probably also limited mobility in ventroflexion and/or lateroflexion in a similar manner to the interspinous ligament that runs along the neural spine in mammals [58]. Although we currently lack detailed information about soft-tissue composition of the sail, future work could explore the implications of different soft-tissue configurations on vertebral motion.

## 5. Conclusion

The synapsid–mammal locomotor transition is one of the most significant events in vertebrate evolution, laying the foundations for the remarkable diversity of locomotor modes that characterize crown Mammalia. In particular, the mammal vertebral column is both morphologically regionalized and functionally diverse, with specialization for different functions in different vertebral modules. Here, we have shed light on the functional changes of the vertebral column during mammalian evolution. We demonstrate that co-option of pre-existing subtle anterior–posterior morphological regions in cynodonts facilitated the evolution of axial rotation in the anterior column, and lateral stabilization in the posterior column, predating the evolution of lumbar sagittal mobility in mammals. This work highlights that evolutionary tinkering with serial homologous elements (e.g. intercentra, orientation of zygapophyses) within specific vertebral regions can lead to innovations in intervertebral joint function. Finally, we have shown that some key aspects of the mammalian functional repertoire originated outside of the crown group, in non-mammalian cynodonts, but that sagittal mobility associated with mammalian-specific gaits, probably appeared later.

## Data Availability

CT scan data for fossil and extant specimens are stored in the repositories of the museum collections in which the specimens are housed and are available from them upon request. Raw data, code for plotting in R and MayaPy files are available in Dryad [59]. Supplementary material is available online [60].
